# Functional disruption of cell wall invertase inhibitor by genome editing increases sugar content of tomato fruit without decrease fruit weight

**DOI:** 10.1038/s41598-021-00966-4

**Published:** 2021-11-02

**Authors:** Kohei Kawaguchi, Rie Takei-Hoshi, Ikue Yoshikawa, Keiji Nishida, Makoto Kobayashi, Miyako Kusano, Yu Lu, Tohru Ariizumi, Hiroshi Ezura, Shungo Otagaki, Shogo Matsumoto, Katsuhiro Shiratake

**Affiliations:** 1 Graduate School of Bioagricultural Sciences, Nagoya University, Chikusa-ku, Nagoya, 464-8601 Japan; 2 Engineering Biology Research Center, Kobe University, Chuo-ku, Kobe, 650-0047 Japan; 3 RIKEN Center for Sustainable Resource Science, Yokohama, 230-0045 Japan; 4 Graduate School of Life and Environmental Sciences, University of Tsukuba, Tsukuba, 305-8572 Japan; 5 Tsukuba Plant Innovation Research Center, University of Tsukuba, Tsukuba, 305-8572 Japan; 6 Faculty of Life and Environmental Sciences, University of Tsukuba, Tsukuba, 305-8572 Japan

**Keywords:** Biotechnology, Plant sciences

## Abstract

Sugar content is one of the most important quality traits of tomato. Cell wall invertase promotes sucrose unloading in the fruit by maintaining a gradient of sucrose concentration between source leaves and fruits, while invertase inhibitor (INVINH) regulates this process. In this study, knock-out of cell wall *INVINH* in tomato (*SlINVINH1*) was performed by genome editing using, CRISPR/Cas9 and Target-AID technologies. Most of the genome-edited lines set higher soluble solid content (SSC) fruit than the original cultivar ‘Suzukoma’, while fruit weight was different among the genome-edited lines. From these genome-edited lines, three lines (193–3, 199–2, and 247–2), whose SSC was significantly higher than ‘Suzukoma’ and fruit weight were almost the same as the original cultivar, were selected. The fruit weight and overall plant growth of the two lines were comparable to those of the original cultivar. In contrast, the fructose and glucose contents in the mature fruits of the two lines were significantly higher than those of the original cultivar. The mature fruits of genome edited line 193–3 showed the highest sugar content, and the fructose and glucose contents were 29% and 36% higher than that of the original cultivar, respectively. Whole genome sequence data showed no off-target mutations in the genome-edited lines. Non-target metabolome analysis of mature fruits revealed that fructose was the highest loading factor in principal component analysis (PCA) between the genome-edited line and the original cultivar, and no unexpected metabolites appeared in the genome-edited line. In this study, we succeeded in producing tomato lines with high sugar content without a decrease in fruit weight and deterioration of plant growth by knock-out of *SlINVINH1* using genome editing technology. This study showed that functional disruption of *SlINVINH1* is an effective approach to produce tomato cultivars with high sugar content.

## Introduction

Consumers in Japan prefer sweet tomatoes, and therefore, tomatoes with high sugar content are a popular quality trait in the Japanese market. High sugar content tomato is becoming popular in other countries as well, and its demand is increasing worldwide. Generally, tomatoes with high sugar content were produced by applying water stress to the tomato plant. However, the major problem of this cultivation is a large decrease in fruit size and yield.

In cultivated tomato, sucrose is produced by photosynthesis in leaves and translocated through the phloem into the fruits, where it is broken down into hexoses, which eventually accumulate in the vacuoles of fruit cells. Two enzymes, sucrose synthase (SuSy, EC 2.4.1.13) and invertase (INV, EC 3.2.1.26), are responsible for the breakdown of sucrose into hexoses^1^. SuSy catalyses the reversible reaction of sucrose + UDP ⇄ UDP-glucose + fructose. UDP-glucose produced from sucrose breakdown is used as a substrate for starch and cellulose syntheses^1,2^. In contrast, INV catalyses the irreversible breakdown of sucrose into glucose and fructose. INV plays important roles in the regulation of osmotic pressure^2^, sucrose unloading from phloem to sink cells, stress responses, and various plant developmental events^3^. Based on their subcellular localisation and pH optima, INVs are classified into cell wall INV, cytoplasmic INV, and vacuolar INV^4^. The optimal pH of cytoplasmic INV is 7.0–7.8; hence, it is also called neutral/alkaline INV^4,5^. In contrast, the optimal pH of cell wall INV and vacuolar INV are ~ 4.5 and 4.5–5.5, respectively; hence, they are also called acid INVs^4^. Vacuolar INV regulates the balance between sucrose and hexose in vacuoles, and the osmotic pressure in cells. Cell wall INV regulates sucrose unloading by maintaining a gradient of sucrose concentration between the source and sink organs through sucrose breakdown in the apoplast of sink organs^3^.

INV is regulated by an invertase inhibitor (INVINH), which binds to the active site (sucrose binding site) of INV^6^. Three decades after the biochemical characterisation of INVINH in the 1960s^7,8^, the first cDNA encoding cell wall INVINH was cloned^9^. Since then, cDNAs encoding INVINH have been reported from various plant species, including tobacco^9^, maize^10^, tomato^11–13^, potato^14^, soybean^15^, and Arabidopsis^16^. Plants have two INVINH: vacuolar INVINH; and cell wall INVINH, which inhibit the activity of vacuolar INV and cell wall INV, respectively. In tomato, Vacuolar INVINH regulates the balance between sucrose and hexose in vacuoles and affects fruit ripening^12^. Conversely, cell wall INVINH regulates the balance between sucrose and hexose in the apoplast, which regulates leaf senescence, cold tolerance, seed weight, and hexose concentration in fruits^11,17^.

Recently, genome editing technologies, which specifically recognise and modify the genome sequence of an organism using artificial nucleases have been developed^18^, such as transcription activator-like effector nuclease (TALEN)^19^, zinc finger nucleases (ZFN)^20^ and CRISPR/Cas9 (clustered regularly interspaced short palindromic repeats/CRISPR associated protein 9)^21^. Among these genome editing technologies, CRISPR/Cas9 has been widely used for genome editing in various organisms, including animals, plants, and microorganisms, because of the ease of changing target sequences and targeting multiple genes.

The CRISPR/Cas9 system recognises and cleaves the complementary genomic DNA through the endonuclease-*Streptococcus pyogenes* Cas9-guide RNA (gRNA) complex^21^. The target sequence can be easily modified by changing the 20 bp gRNA sequence in the CRISPR/Cas9 system. Cleaved double-stranded DNA is repaired by non-homologous end joining^21^; however, repair errors such as deletion, insertion, or substitution of nucleotides can occur during this process. CRISPR/Cas9 system is used to alter or disrupt promoter or gene function not only for research but also for plant breeding.

Furthermore, nuclease deficient Cas9 (dCas9) was developed and used with gRNA to recognise specific DNA sequences without inducing DNA breakage. CRISPR imaging, which involves imaging specific loci using dCas9 fused with fluorescent protein^22,23^ and regulation of gene expression using dCas9 fused with transcriptional activator or repressor^24,25^ have been reported. Target-AID (Target Activation Induced Cytidine Deaminase) is an application^26^ which uses cytidine deaminase PmCDA1 from *Petromyzon marinus* fused with dCas9 or nCas9 (nickase Cas9 which has single-strand DNA cleavage activity). Target-AID using dCas9 causes highly efficient and accurate substitution of cytosine to thymine in the target site by PmCDA1. Target-AID using nCas9 not only caused base substitution but also deletion and insertion in the target site at high efficiency and has demonstrated the feasibility of genome editing for crop improvement^27^.

A previous study reported that the knock-down of cell wall *INVINH* by RNAi technology in tomato increased the activity of INV and sucrose unloading in fruit by maintaining a gradient of the sucrose concentration between source leaves and fruit, resulting in an increased sugar content in fruits^11^. In the present study, we aimed to increase the sugar content of tomato fruits by knock-out of the cell wall *INVINH* using CRISPR/Cas9 and Target-AID technology.

## Results and discussions

### Production of cell wall *INVINH* knock-out tomato

**Figure 1:**
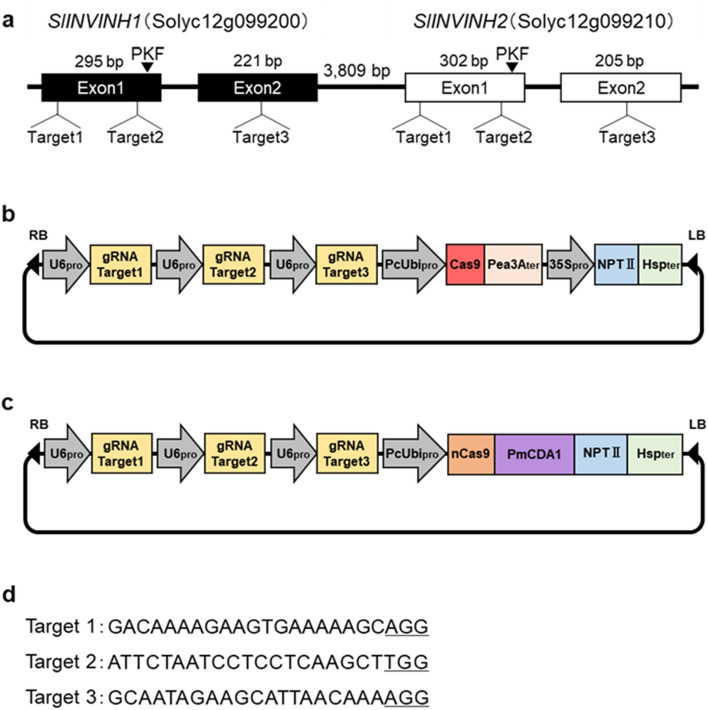
Schematic diagram of CRISPR/Cas9 and Target-AID target sites in *SlINVINHs* and vector construction. (**a**) Position of *SlINVINH1*, *SlINVINH2* and three guide RNA targets in *SlINVINHs.* Boxes indicate exons. The black arrowhead indicates the PKF (proline-lysine-phenylalanine) motif. (**b**) Schematic map of the CRISPR/Cas9 vector, and (**c**) Target-AID vector. RB: right border. LB: left border. U6pro: AtU6-26 promoter. gRNA: guide RNA. PcUbi: *Petroselinum crispum* ubiquitin promoter. Cas9: *Streptococcus pyogenes* Cas9 gene. nCas9: nickase Cas9 gene. PmCDA1: *Petro myzontiformes* cytidine deaminase 1 gene. PeathreeAter: 3A terminator. 35S pro: *Cauliflower mosaic virus* 35S promoter. NPTII: Kanamycin resistance gene. Hspter: heat shock protein gene terminator. (**d**) Sequences of the three targets. The PAM sequences (NGG) are underlined.

Two genes encoding cell wall INVINHs, *SlINVINH1* (Solyc12g099200) and *SlINVINH2* (Solyc12g099210), are present in the genome of tomato *Solanum lycopersicum* ‘Heinz 1706’ (Sol Genomics Network, https://solgenomics.net/). *SlINVINH1* and *SlINVINH2* have high sequence homology with each other at both the nucleotide and amino acid levels, and both genes consist of two exons and one intron. *SlINVINH1* and *SlINVINH2* are tandemly present in the tomato genome, and the distance between them is only 3,809 bp (Fig. 1a). Therefore, these two genes may have appeared due to gene duplication. The expression level of *SlINVINH2* shown by the Tomato eFP browser (http://bar.utoronto.ca/efp2/Tomato/Tomato_eFPBrowser2.html, ^28^) was lower in all organs and tissues. A study reported that specific silencing of *SlINVINH1* increased hexose levels in tomato fruits^11^. Therefore, we considered that *SlINVINH2* is a pseudogene or does not play an important role in tomato fruit. To be sure, we selected three consensus sequences between *SlINVINH1* and *SlINVINH2* as target sequences for CRISPR/Cas9 and Target-AID, expecting knock-out of not only *SlINVINH1* but also of *SlINVINH2* (Fig. 1a,d). The efficiency of mutations by CRISPR/Cas9 was reported to depend on the target sequence^29^. In this study, two target sequences in the first exon (Target 1 and Target 2) and one target sequence in the second exon (Target 3), a total of three target sequences were used for multiplex genome editing (Fig. 1a,d). The proline-lysine-phenylalanine (PKF) motif in INVINH competes with sucrose in the binding site of INV; thus, the PKF motif in INVINH is important for the inhibition of INV activity^6^. Target 1 and Target 2 exist upstream of the PKF motif (Fig. 1a); therefore, the mutation in Target 1 or Target 2 can lead to loss of function of INVINH.

To produce *SlINVINH* knock-out tomato lines, cotyledon explants of tomato ‘Suzukoma’ were transformed with *Agrobacterium tumefaciens* GV2260 harbouring CRISPR/Cas9 vector (Fig. 1b, Fig. S1) or Target-AID vector (Fig. 1c, Fig. S2). Gene transformation in the differentiated shoots from callus was checked by PCR, and only transformants were selected. Tomato frequently produces polyploid plants during de-differentiation and differentiation processes. Therefore, ploidy of the transformants was confirmed, and only diploid plants were selected. Furthermore, considering the selection efficiency of vector-free plants (null segregant plants) in the next generation, the copy number of the transformed gene was determined by qPCR, and only transformants with single copy transformed gene were selected.

### Mutation patterns in *SlINVINH1* by CRISPR/Cas9 or Target-AID

The mutation pattern of *SlINVINH1* by CRISPR/Cas9 or Target-AID was confirmed by direct sequencing of *SlINVINH1* in the transformants. Therefore, five transformants by CRISPR/Cas9 and eight transformants by Target-AID, which have nucleotide(s) insertion or deletion in the exon of *SlINVINH1*, were obtained. These transformants (T_0_ generation) were self-pollinated and T_1_ seeds were obtained. Among the T_1_ plants, null segregants with homozygous mutations in *SlINVINH1* were selected.

**Figure 2:**
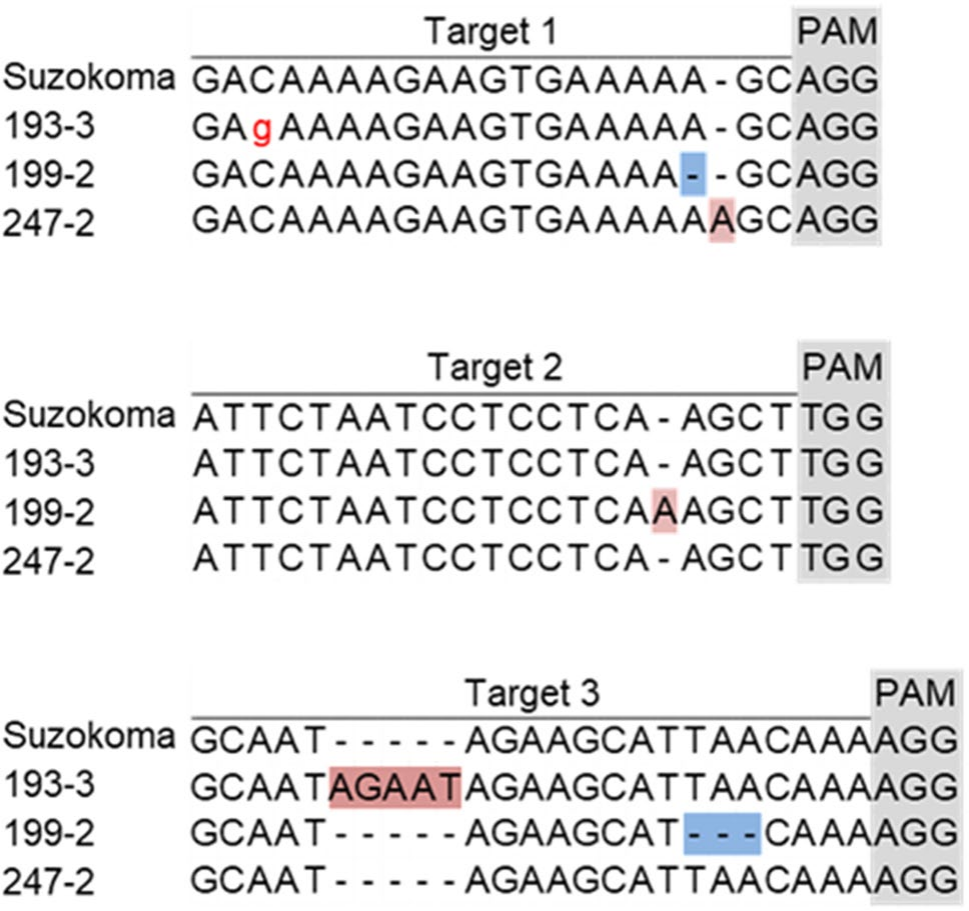
Mutations in *SlINVINH1* of the genome edited lines. Target sequence in original cultivar ‘Suzukoma’ is shown at the top in each panel and the sequence in three genome edited lines (193–3, 199–2, and 247–2) are shown below. Grey shade indicates PAM sequence. Red shade, blue shade or red small letter show insertion, deletion, or substitution of nucleotide(s), respectively.

In the CRISPR/Cas9 genome-edited lines, nucleotide(s) insertion or deletion at 3 bp downstream from the Proto-spacer Adjacent Motif (PAM) sequence occurred frequently, such as the adenine insertion 3 bp downstream of the PAM sequence of Target 1 in line 247–2 (Fig. 2). This trend was consistent with the report by Jinek et al.^21^, which shows that the mutations by CRISPR/Cas9 occurred frequently around 3 bp downstream of the PAM sequence.

In contrast, in the genome-edited lines by Target-AID using nCas9, insertion or deletion at 3 bp downstream of the PAM sequence, but also substitution of cytosine around 18 bp downstream of PAM sequence occurred frequently, such as the substitution from cytosine to guanine of Target 1 in line 193–3 (Fig. 2). This trend was consistent with the report by Nishida et al.^26^, where mutations by Target-AID using nCas9 occurred frequently around 3 bp downstream of the PAM sequence and at 15–19 bp downstream from the PAM sequence.

### Phenotyping of the* SlINVINH1* knock-out tomato

In 13 of the genome-edited lines (T_1_ generation), which are null segregants with homozygous mutations in *SlINVINH1,* fruit weight and soluble solid content (SSC) of mature fruit were measured. Although the SSC average was higher in the genome-edited lines than the original cultivar ‘Suzukoma’ (Fig. S3a), fruit weight differed among the genome-edited lines (Fig. S3b). From the 13 genome-edited lines, three lines (193–3, 199–2, and 247–2), whose SSC average was higher than ‘Suzukoma’ and fruit weight almost equal to ‘Suzukoma’ (Fig. S3) were selected for further analysis.

**Figure 3:**
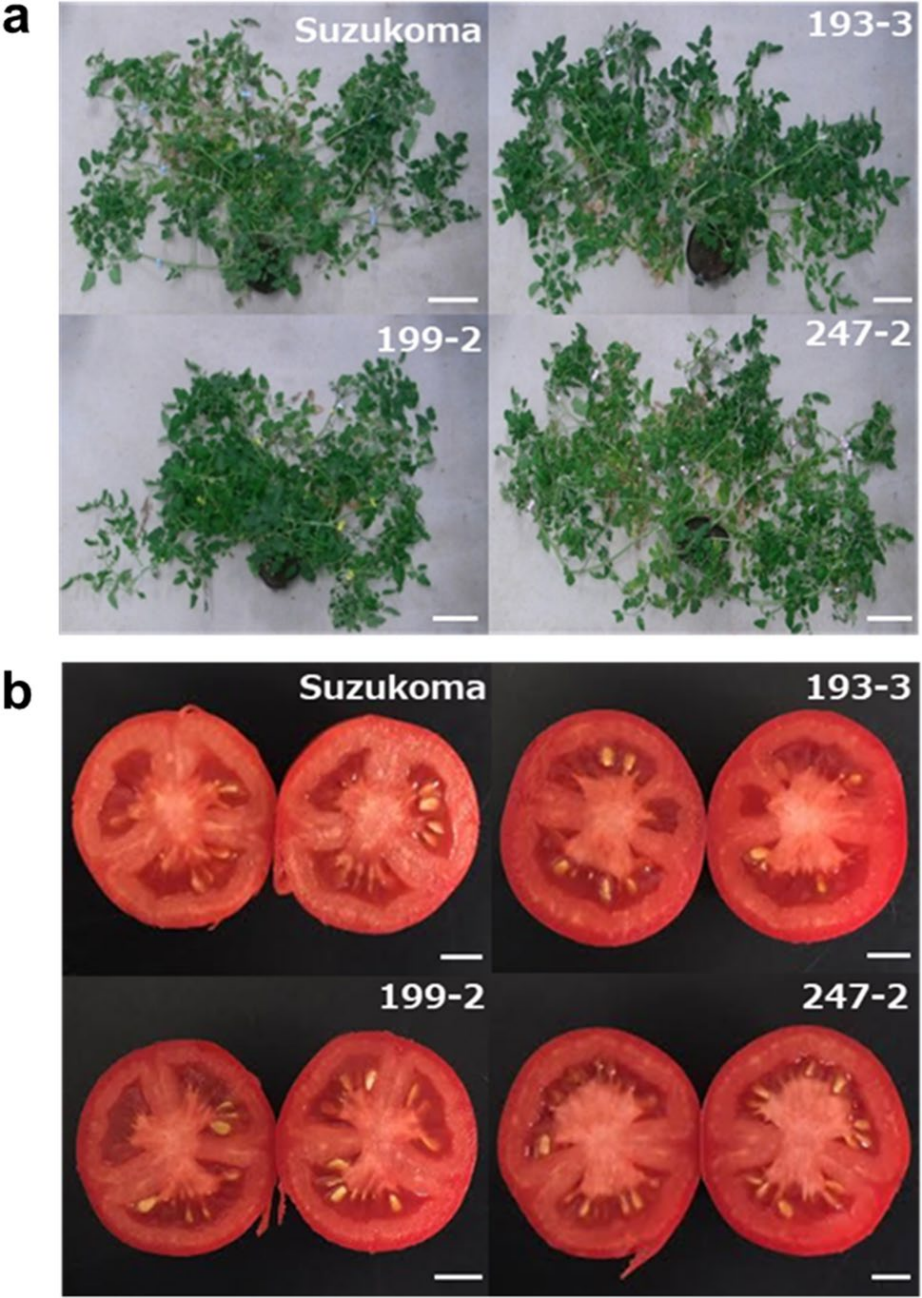
Phenotypes of the *SlINVNH1* genome edited lines. (**a**) Visual vegetative phenotype of original cultivar ‘Suzukoma’ and the *SlINVINH1* genome edited lines (193–3, 199–2, and 247–2). Scale bar = 15 cm. (**b**) Fruits at mature stage from ‘Suzukoma’ and the *SlINVINH1* genome edited lines. Scale bar = 1 cm.

**Figure 4:**
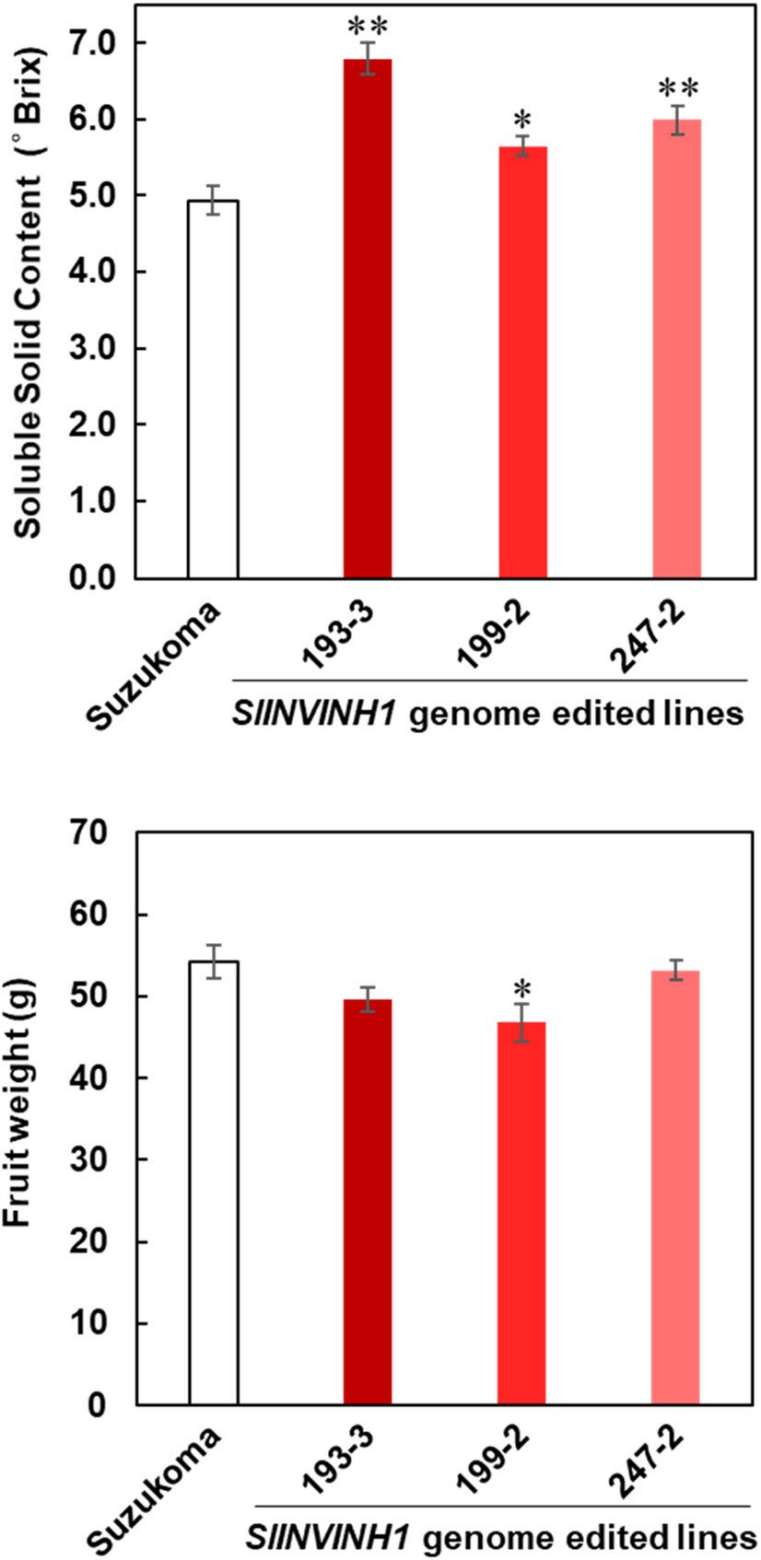
SSC and weight of fruits from the *SlINVINH1* genome edited lines. (**a**) SSC of fruits at mature stage from original cultivar ‘Suzukoma’ and the *SlINVINH1* genome edited lines (193–3, 199–2, and 247–2). Error bars indicate standard error for 15 fruits from 5 plants. An asterisk indicates a significant difference (Dunnett’s test, *P < 0.05; **P < 0.01). (**b**) Fruit weight at mature stage from original cultivar ‘Suzukoma’ and the *SlINVINH1* genome edited lines (193–3, 199–2, and 247–2). Error bars indicate standard error for 15 fruits from 5 plants. An asterisk indicates a significant difference (Dunnett’s test, *P < 0.05).

Overall plant growth and visual vegetative phenotype of the three selected genome-edited lines (T_2_ generation) showed no difference compared with the original cultivar (Fig. 3). On comparison with ‘‘Suzukoma’, lines 193–3, 199–2, and 247–2 showed 37.6%, 14.5%, and 21.4% significantly higher SSC, respectively (Fig. 4a), while no significant difference, 13.7% significantly lower, and no significant difference in fruit weight was observed, respectively (Fig. 4b). Thus, we succeeded in producing tomato lines with high sugar content (193–2 and 247–2) without a decrease in fruit weight and deterioration of plant growth. These results indicate that knock-out of *SlINVINH1* by genome editing is an effective approach to produce high-sugar content tomato cultivars.

Mutation patterns of *SlINVINH1* and *SlINVINH2* in the three genome-edited lines (193–3, 199–2, and 247–2) are shown in Fig. 2 and Figure S4, respectively. As described above, frameshift mutations presumably lead to loss of function in *SlINVINH1* in the three lines (Fig. 2), and in *SlINVINH2* in lines 199–2 and 247–2 (Fig. S4). Only base substitution occurred in *SlINVINH2* in line 193–3, in which SSC of mature fruit was the highest among the three lines. These results reinforce our hypothesis that *SlINVINH2* does not play an important role, and the knock-out of only *SlINVINH1* is sufficient to increase the sugar content of tomato fruit.

**Figure 5:**
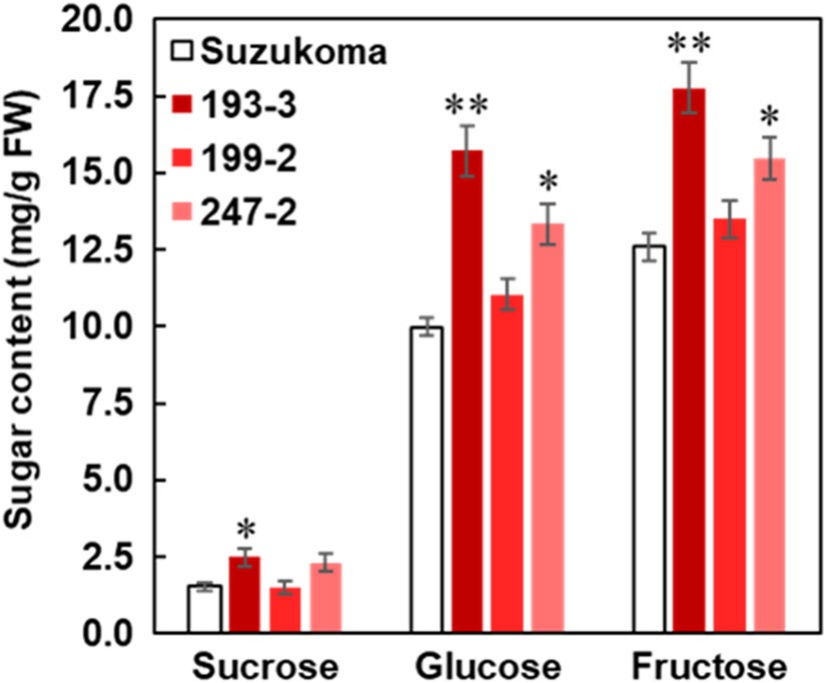
Sugar content of fruits from the *SlINVINH1* genome edited lines. Sucrose, glucose and fructose content in mature fruits from original cultivar ‘Suzukoma’ and the *SlINVINH1* genome edited lines (193–3, 199–2, and 247–2). Error bars indicate standard error for 15 fruits from 5 plants. An asterisk indicates a significant difference (Dunnett’s test, *P < 0.05; **P < 0.01).

Knock-down of *SlINVINH1* by RNAi technology in tomato increases the hexose content of fruits^11^. Among the three genome-edited lines (193–3, 199–2, and 247–2), two lines (193–3 and 247–2) showed significantly higher fructose and glucose content in mature fruits than the original cultivar ‘Suzukoma’ (Fig. 5). The mature fruits of line 193–3 showed the highest sugar content in mature fruit with fructose and glucose contents 29% and 36% higher than ‘Suzukoma’, respectively (Fig. 5). The increase in hexose content of the *SlINVINH1* genome-edited lines might have been caused by higher sucrose hydrolysis in the apoplast of fruits by retention of high cell wall INV activity, as reported in earlier studies^11^.

### Transcript level of *SlINVINH1* and mutation in the *SlINVINH1* transcript

To determine the transcript level of *SlINVINH1* in the genome-edited lines, quantitative RT-PCR analysis of *SlINVINH1* was performed using RNA from mature fruit, which showed high *SlINVINH1* expression. Therefore, the transcript level of *SlINVINH1* decreased in the genome-edited lines compared to the original cultivar ‘Suzukoma’. However, the difference in *SlINVINH1* transcript levels between the genome-edited lines and ‘Suzukoma’ was not significant (Fig. S5). This was expected because the target sequences of genome editing were chosen in the coding region of *SlINVINH1*, but not in the promoter region.

To prove that the transcript of *SlINVINH1* lost its function in the genome-edited lines, the sequence of the *SlINVINH1* transcript was determined. Similar patterns of insertions, deletions, and substitutions, which were found in the genome of the genome-edited lines (Fig. 2), were found in the *SlINVINH1* transcripts of the genome-edited lines (Fig. S6), indicating that *SlINVINH1* mRNA lost its function. This result supported that the phenotypic effect was due to genome editing of the *SlINVINH1* gene.

### Off-target mutation analysis of the *SlINVINH1* knock-out tomato

Genome editing by CRISPR/Cas9 can induce off-target mutations as well as in the sequences similar to those of the target sequence^29^. Therefore, to confirm the presence of any off-target mutations in the genome-edited lines (193–3, 199–2, and 247–2), whole genome sequencing was performed and the sequences with mismatches of three bases or less with the target sequences (Target 1, Target 2, and Target 3) in the tomato genome were checked. Therefore, no off-target mutations were found in the *SlINVINH1* genome-edited lines in the checked sequences (Table S1).

### Non-target metabolome analysis of the *SlINVINH1* knock-out tomato

To evaluate the fruit quality of the *SlINVINH1* knock-out tomato, non-target metabolome analysis was performed using GC-TOF/MS and fruit metabolites between the *SlINVINH1* genome editing line 193–3 and the original cultivar ‘Suzukoma’ were compared. Metabolome analysis revealed 148 metabolite peaks, including unidentified peaks. Among them, 76 metabolites were identified, and 19 metabolite levels, including fructose, glucose, and sucrose levels, were significantly different between the genome-edited line and the original cultivar ‘Suzukoma’. The relative abundance of the 19 metabolites were 0.51–4.01 fold-change (Table S2). Among the 16 metabolites, excluding fructose, glucose, and sucrose, 10 metabolites significantly increased and 6 metabolites significantly decreased in the genome-edited line compared to the original cultivar (Table S2). Metabolites, such as Trehalose, glucose-6-phosphate, isomaltose, and myo-inositol were found to increase in the genome-edited line. These metabolites are synthesised from glucose. Therefore, the higher glucose levels may have increased the biosynthesis of these metabolites in the genome-edited line (Fig. 5). Two amino acids (asparagine and glutamine) and one organic acid (malic acid) were found to be the decreased metabolites in the genome-edited line. Although the reason for the decrease in these metabolites is unclear, the taste of tomato fruits may be affected because these metabolites are associated to taste^30,31^. No unexpected metabolite peak appeared in the genome-edited line, indicating that no unexpected metabolite accumulated in the fruits of the genome-edited line.

**Figure 6:**
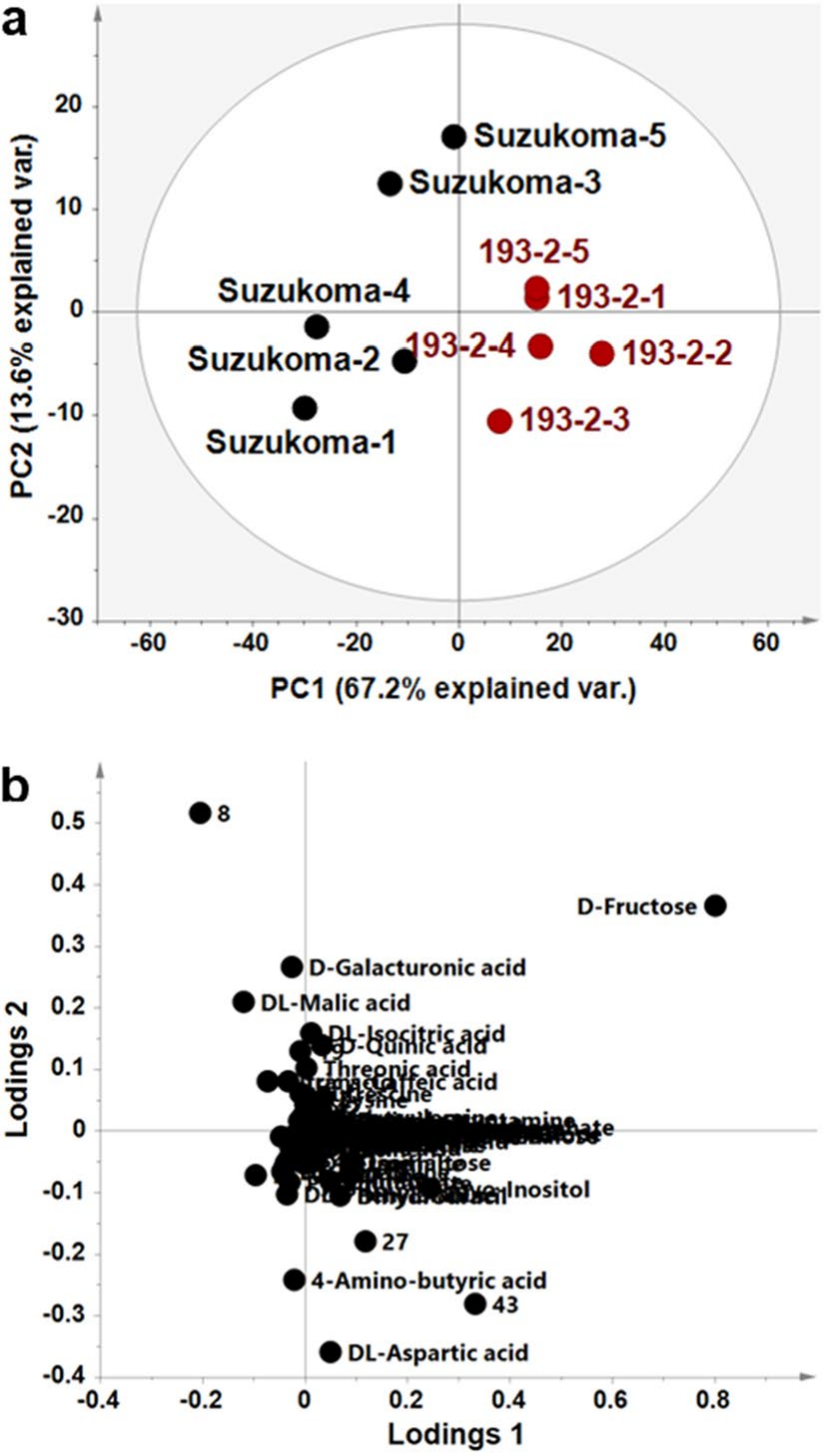
Principal component analysis (PCA) of metabolites in mature fruits from the *SlINVINH1* genome edited line. Metabolite levels in mature fruits obtained from original cultivar ‘Suzukoma’ and the *SlINVINH1* genome edited line (193–3) were determined by GC-TOF/MS. (**a**) Score scatter plot of the PCA. Black symbol indicates ‘Suzukoma’ and red symbol indicate the *SlINVINH1* genome edited lines (*n* = 5 for each sample). (**b**) Loading scatter plot of the PCA analysis (*n* = 148 metabolite peaks). Numbers indicate unknown peak of metabolites.

The metabolome data obtained were subjected to variable standardisation and principal component analysis (PCA). In the PCA score scatter plot, *SlINVINH1* genome-edited line 193–3 and original cultivar ‘Suzukoma’ were separated in the direction of the first principal component axis (Fig. 6a). In the PCA loading scatter plot, fructose was found to have the highest loading factor (80.2%) and the other metabolites had a low loading factor (less than 30%) in the first principal component separation (Fig. 6b). These results show that fructose was the highest contributing factor for principal component separation between *SlINVINH1* genome edited line 193–3 and ‘Suzukoma’.

### Approaches to produce high sugar content tomato

For the production of tomato with high sugar content, two approaches, including breeding and cultivation, have been applied.

Under cultivation approach, water stress and salt stress have been applied to tomato cultivation to increase starch accumulation in fruit at an early developmental stage^32^ and to induce the concentration effect of metabolites in fruit at a later developmental stage^33^. However, the application of water stress or salt stress induces cracking of fruit and blossom-end rot. Therefore, a high cultivation technique to control water status and labour for this control is necessary for such cultivation. The most serious disadvantage of water stress or salt stress in cultivation to produce high sugar content tomato is a large decrease in fruit weight^34^, with more than 50% decrease in some cases. However, in the case of *SlINVINH1* genome-edited tomato, no high cultivation technique and labour are necessary, and there was no decrease in fruit weight (Fig. 4b).

Cross breeding and mutation breeding have been performed as breeding approaches. By gamma ray irradiation, the mutant of tomato dwarf cultivar ‘Micro-Tom’, whose SSC in fruit is four degrees higher than the original cultivar, was screened^35^. Among the introgression lines of *S. lycopersicum* ‘M82’ containing chromosome fragments of the wild tomato species *S. pennellii,* the lines with higher sugar content in the fruit were screened^35^. Later, cell wall INV^37,38^ and ADP-glucose pyrophosphorylase (AGPase)^39^ from *S. pennellii* were identified as the genes responsible for high sugar content of the introgression lines.

Transgenic technology has also been used to produce tomato cultivars with high sugar content. Introduction of the apple hexose transporter gene (*MdHT2.2*)^40^ or peach sucrose non-fermenting-1-related protein kinase 1 gene (*SnRK1*)^41^ into tomato increased the sugar content of fruit. Overexpression of the tomato phosphoenolpyruvate carboxykinase gene (*SlPEPCK*)^42^ or auxin response factor 10 gene (*SlARF10*)^43^ also promoted sugar accumulation in fruits.

### Advantages of the *SlINVINH1* knock-out tomato

Jin et al.^11^ reported an increase in the hexose content of tomato fruit by knock-down of *SlINVINH1* using RNAi technology. In this study, we succeeded in producing tomatoes with high sugar content by knock-out of *SlINVINH1* using genome editing technology, without deterioration in plant growth and decrease in fruit weight. The *SlINVINH1* knock-out tomato can produce high sugar content fruit without a high cultivation technique that needs control water and labour. These characteristics of the *SlINVINH1* knock-out tomato show that it can be a useful high sugar content tomato cultivar, and the knock-out of *SlINVINH1* by genome editing technology is an effective approach to produce them.

To sell genome-edited crops on the market, it is necessary to provide sufficient and reliable information to consumers. In this study, we confirmed that the *SlINVINH1* knock-out tomato lines are transgene-free and free of off-target mutations using whole genome sequencing. In addition, to confirm metabolome equivalence between the genome-edited line and the original cultivar, non-target metabolome analysis was performed. Therefore, although the levels of 19 metabolites, including fructose, glucose, and sucrose, were significantly different between the genome-edited line and the original cultivar ‘Suzukoma’, the differences were not so large, and no unexpected metabolite accumulation appeared in the genome-edited line. These data may help consumers accept genome-edited tomatoes.

In this study, we succeeded in producing high sugar content tomato by knock-out of cell wall *INVINH* using genome editing technology. Cell wall *INVINH* is highly conserved among various plant species. Hence, knock-out of cell wall *INVINH* by genome editing can increase the sugar content of various other fruits, and this technology is expected to be widely used in breeding to produce high sugar content fruit crops.

## Materials and methods

All experimental research on plants complied with relevant institutional, national, and international guidelines and legislation.

### Plant material

Tomato (*Solanum lycopersicum* ‘Suzukoma’) was grown in the greenhouse at Nagoya University (Nagoya, Japan). Peat Pot P (Hokkaido Peat Moss, Saitama, Japan) was used as the culture soil. Water containing liquid fertiliser (Otsuka House No. 1 and No. 2, Otsuka Chemical, Osaka, Japan) was applied to plants by bottom irrigation. Five plants from each line and three fruits from each plant were used for phenotyping.

### Vector construction

Three consensus sequences with the PAM sequence (5'-NGG-3') in the exons of *SlINVINH1* and *SlINVINH2* as target sequences of CRISPR/Cas9 and Target-AID were selected (Fig. 1a,d). These three target sequences (Target1-3) were cloned downstream of the Arabidopsis U6-26 promoter (U6pro) in the CRISPR/Cas9 (Fig. 1b, Fig. S1), or Target-AID vectors (Fig. 1c, Fig. S2). These vectors were transformed into *A. tumefaciens* GV2260 by electroporation and used for the genetic transformation of tomatoes.

### Genetic transformation of tomato

Transformation of tomatoes was performed by modifying the method described by Amemiya et al.^44^. Cultured *A. tumefaciens* harbouring CRISPR/Cas9 (Fig. 1b, Fig. S1) or Target-AID vectors (Fig. 1c, Fig. S2) was suspended in liquid MS medium containing 100 µM acetostringone and 10 µM 2-mercaptoethanol. Cotyledon explants from tomato seedlings at ca. 10 days after sowing were immersed in the *A. tumefaciens* suspension for 10 min. The explants were co-cultured with *A. tumefaciens* on MS basal medium (MS medium containing 2% (w/v) sucrose and 0.8 (w/v) agar, pH 5.8) containing 50 µM acetosyringone in the dark. The explants were then transferred to the callus induction medium (MS basal medium containing 50 µg/mL kanamycin, 375 µg/mL augmentin, and 1.5 µg/mL *trans*-zeatin) for selection of transformants, callus induction, and sterilisation of *A. tumefaciens*. After shoot induction from the callus, the calluses were transferred to shoot elongation medium (basal MS medium containing 50 µg/mL kanamycin, 375 µg/mL augmentin, and 1 µg/mL *trans*-zeatin) for selection of transformants, shoot elongation, and sterilisation of *A. tumefaciens*. After shoot elongation, the shoots were transferred to the root induction medium (MS medium containing 2% (w/v) sucrose, 50 µg/mL kanamycin, 375 µg/mL augmentin, and 0.3% (w/v) gelrite, pH 5.8) for selection of transformants, root induction, and sterilisation of *A. tumefaciens*. After rooting, the shoots with roots were transferred to the soil and acclimated. Explants, calluses, and shoots were grown under 16 h light/8 h dark at 25 °C and transferred to new medium every 2 weeks.

### Ploidy analysis

Nuclei were extracted from young leaves by chopping and fluorescently stained with CyStain UV Precise P (Sysmex, Hyogo, Japan). Fluorescence was detected using a PA type Ploidy Analyser (Partec, Görlitz, Germany). Ploidy of transformants was confirmed by comparison with the peak of the original cultivar ‘Suzukoma’.

### Confirmation of genetic transformation and copy number of the transformed gene

Genomic DNA was extracted from young leaves according to the method described in Edwards et al.^45^. The DNA precipitate was added to 200 µL isopropanol and centrifuged at 18,000 × *g* for 5 min at 20 °C, 200 µL 70% (v/v) ethanol was added, and the mixture was centrifuged at 18,000 × *g* for 5 min at 20 °C for purification. The DNA precipitate was suspended in TE buffer (1 mM EDTA, 10 mM Tris–HCl, pH 8.0).

Genetic transformation was confirmed by PCR amplification of the *Cas9* and *PcUbi* promoter regions (Fig. 1b,c) with the primers under the conditions described in Table S3.

The copy number of the transformed gene was confirmed by quantitative PCR (qPCR) amplification of the *Cas9* fragment (Fig. 1b,c) with the primers under the conditions described in Table S4. qPCR analysis was performed using the Step One Plus Real-Time PCR System (Applied Biosystems, Massachusetts, USA) and TB Green Premix Ex Taq II (Takara Bio, Shiga, Japan). Copy number was estimated by calculating the relative value of *Cas9* to *LAT52*, which was reported to be a single copy gene in the tomato genome^46^.

### Confirmation of mutation of *SlINVINH1* in genome and transcript

The fragments covering the three target sequences in *SlINVINH1* (Fig. 1a) were amplified by PCR using genomic DNA or cDNA prepared for quantitative RT-PCR analysis as described below. The primers and PCR conditions for genomic DNA or cDNA are described in Table S5 and S6, respectively. The obtained PCR product was purified using ExoSAP-IT for PCR Product Cleanup kit (Applied Biosystems). Sequencing reaction was performed using the Big Dye Terminator v3.1 Cycle Sequence Kit (Thermo Fisher Scientific, Massachusetts, USA) and, DNA sequencing was performed using a 3730xl DNA Analyser (Thermo Fisher Scientific).

### Quantitative RT-PCR analysis of *SlINVINH1*

Total RNA was extracted from the 100 mg pericarp of mature fruit using the innuPREP Plant RNA Kit (Analytik Jena, Jena, Germany), according to the manufacturer’s protocol. cDNA was synthesised from total RNA using the PrimeScript RT reagent kit (Takara Bio). Quantitative PCR 0.4 μL of ROX II dye (50X) and 7 μL of sterilised water. The primers and PCR conditions are described in Table S7. Amplification was performed using the Step One Plus Real-Time PCR System (Applied Biosystems). Relative expression levels were normalised to *SlUBQ3* as the reference gene and calculated using the standard curve method. Three biological replicates of each genome-edited line were analysed.

### Measurement of SSC and sugar content

SSC of the pericarp of mature fruit was measured using a Digital Refractometer PR-101 (ATAGO, Tokyo, Japan). Fifteen fruits from five plants of each line were used for the measurement.

Sugar content was measured as described below: 800 µL of ethanol and 50 µL of 1 M mannitol were added to 200 mg pericarp of mature fruit and incubated at 80 °C for 30 min, then centrifuged at 21,000 × *g* for 5 min at 20 °C. The supernatant was dried under vacuum and suspended in water.

The suspension was passed through a Sep-Pak light C18 (Waters, Massachusetts, USA) and 0.45 µm microfilter (ADVANTEC, Tokyo, Japan) and used as a sample for high-performance liquid chromatography (HPLC) analysis. A Chromaster UV–VIS system (Hitachi High-Tech Science, Tokyo, Japan) and Shodex Sugar SP0810 (SHOWA DENKO, Tokyo, Japan) were used for HPLC analysis. Column temperature was maintained at 80 °C. A refractive index detector (Refractive Index Detector RI-3H, Japan Analytical Industry, Tokyo, Japan) was used.

### Whole genome sequencing and off-target mutation analysis

Genomic DNA was extracted from young leaves using a DNeasy Plant Kit (QIAGEN, Hilden, Germany). The DNA library was prepared using the Illumina TruSeq Library Construction Kit (Illumina, California, USA), and sequencing was performed using Hiseq 2500 (Illumina). Adapter sequences and low-quality sequences were removed from raw sequence data by Cutadapt3 ver1.1 (https://cutadapt.readthedocs.io/en/stable/) and Trimmomatic ver0.32 (http://www.usadellab.org/cms/?page=trimmomatic), respectively. The sequence data was mapped on the *S. lycopersicum* ‘Heinz 1706’ genome sequence (SL2.50, ftp://ftp.solgenomics.net/tomato_genome/assembly/build_2.50/) using the short reads mapping program BWA ver0.7.10 (http://bio-bwa.sourceforge.net/). The resulting mapping data were realigned using SAMtools ver1.2 (http://www.htslib.org/man/samtools/) and GATK Lite version 2.3.0 (https://software.broadinstitute.org/gatk/) to improve the mapping accuracy. PCR duplicates were removed using Picard ver1.133 (http://broadinstitute.github.io/picard/).

Off-target sites with a mismatch of three bases or less were searched using CRISPR RGEN Tools Cas-OFFinder (http://www.rgenome.net/cas-offinder/, ^47^). The presence or absence of mutations at the off-target site was confirmed by IGV viewer (http://software.broadinstitute.org/software/igv/).

### Metabolome analysis

Metabolome analysis was performed according to^48–51^. Five fruits from each line were used for the analysis. Metabolites in the pericarp of mature fruit were crushed and then extracted by adding methanol/chloroform/water (3:1:1, v/v/v) containing 10 stable isotope references to a concentration of 25 mg FW/mL. After centrifugation, the supernatant was decompressed and dried. To derivatise metabolites, 30 µL of 20 mg/mL of methoxylamine hydrochloride in pyridine solution was added and kept at room temperature for 24 h. Subsequently, to trimethylated, 30 µL of *N*-methyl-*N*-trimethylsilyltrifluoro acetamide was added and incubated at 37 °C for 1 h. Prior to analysis, 30 uL of *n*-heptane was added to each sample (18.5 µg FW equivalent per µL of the solution). The derivatised metabolites were analysed by Agilent 6890 N GC gas chromatography (Agilent Technologies, California, USA) with a Rxi-5 Sil MS column (30 m, 0.25 mm, 0.25 µm, RESTEK, Pennsylvania, USA) and a time-of-flight mass spectrometer (Pegasus IV TOF MS, LECO, Michigan, USA). Raw data obtained from GC-TOF/MS analysis were transferred from the ChromaTOF software in NetCDF format to MATLAB software 7.13 (https://jp.mathworks.com/products/matlab.html, MathWorks, Massachusetts, USA). The chromatograms were pre-processed using the high-throughput data analysis method^48^ and then normalised using the cross-contribution compensating multiple standard normalisation algorithm^51^. The intensity data of each metabolite mass spectrum were subjected to variable standardisation by pareto scaling. Principal component analysis (PCA) was performed using SIMCA ver15.0 (Sartorius, Göttingen, Germany).

### Statistical analysis

Multiple tests were performed by R statistical software version 4.0.4 (R Project for Statistical Computing, https://www.r-project.org/) within RStudio statistical software version 1.4.1106 (RStudio, https://www.rstudio.com/) using two-tailed Dunnett’s test and *P* < 0.05 or *P* < 0.01 was considered as significant difference.

t test was performed by Microsoft EXCLE 2019 and *P* < 0.05 was considered as significant difference.

## Supplementary Information

Supplementary Information 1.

Supplementary Information 2.

Supplementary Information 3.

Supplementary Information 4.

Supplementary Information 5.

Supplementary Information 6.

Supplementary Information 7.

Supplementary Information 8.
