# Supplementary material for: Functional disruption of cell wall invertase inhibitor by genome editing increases sugar content of tomato fruit without decrease fruit weight
Source: Sci Rep. 2021 Nov 2;11:21534. doi: 10.1038/s41598-021-00966-4 (PMC8563804; doi:10.1038/s41598-021-00966-4)
Supplement: Supplementary file 8 — Supplementary Information 8. [file 41598_2021_966_MOESM8_ESM.docx]

**Functional Disruption of Cell Wall Invertase Inhibitor by Genome Editing Increases Sugar Content of Tomato Fruit without Decrease Fruit Weight**

Kohei Kawaguchi^1^, Rie Takei-Hoshi^1^, Ikue Yoshikawa^1^, Keiji Nishida^2^, Makoto Kobayashi^3^, Miyako Kusano^3, 4, 5^, Yu Lu^6^, Tohru Ariizumi^5, 6^, Hiroshi Ezura^6^, Shungo Otagaki^1^, Shogo Matsumoto^1^ and Katsuhiro Shiratake^1*^

^1^Graduate School of Bioagricultural Sciences, Nagoya University, Chikusa-ku, Nagoya 464-8601, Japan

^2^Engineering Biology Research Center, Kobe University, Chuo-ku, Kobe 650-0047, Japan

^3^RIKEN Center for Sustainable Resource Science, Yokohama 230-0045, Japan

^4^Graduate School of Life and Environmental Sciences, University of Tsukuba, Tsukuba 305-8572, Japan

^5^Tsukuba Plant Innovation Research Center, University of Tsukuba, Tsukuba 305-8572, Japan

^6^Faculty of Life and Environmental Sciences, University of Tsukuba, Tsukuba 305-8572, Japan

*Corresponding author (email: shira@agr.nagoya-u.ac.jp)

**
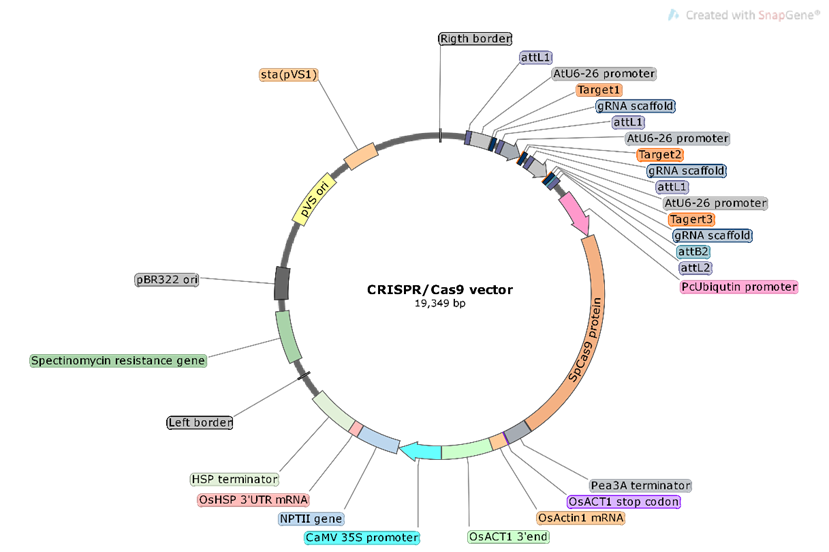
**

**Supplementary Figure S1. Schematic diagram of CRISPR/Cas9 vector construction.**

**
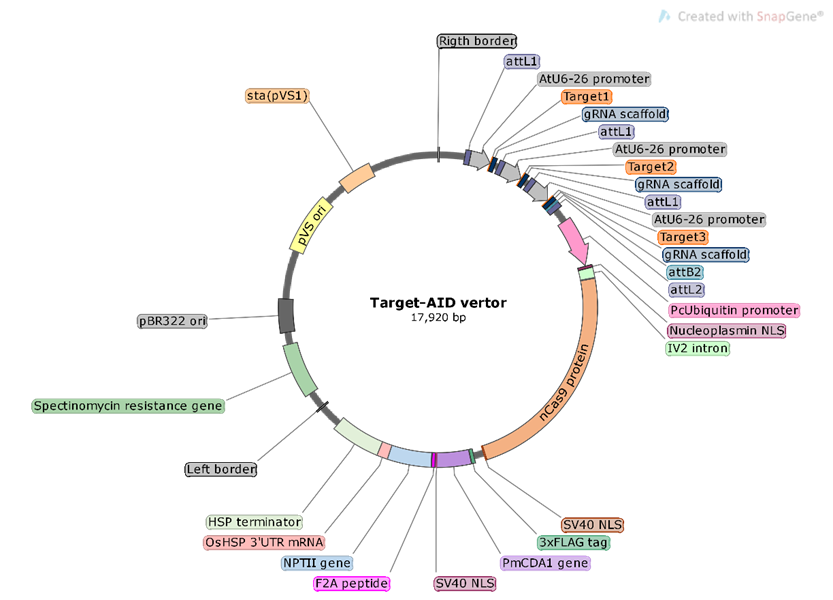
**

**Supplementary Figure S2. Schematic diagram of Target-AID vector construction.**

**
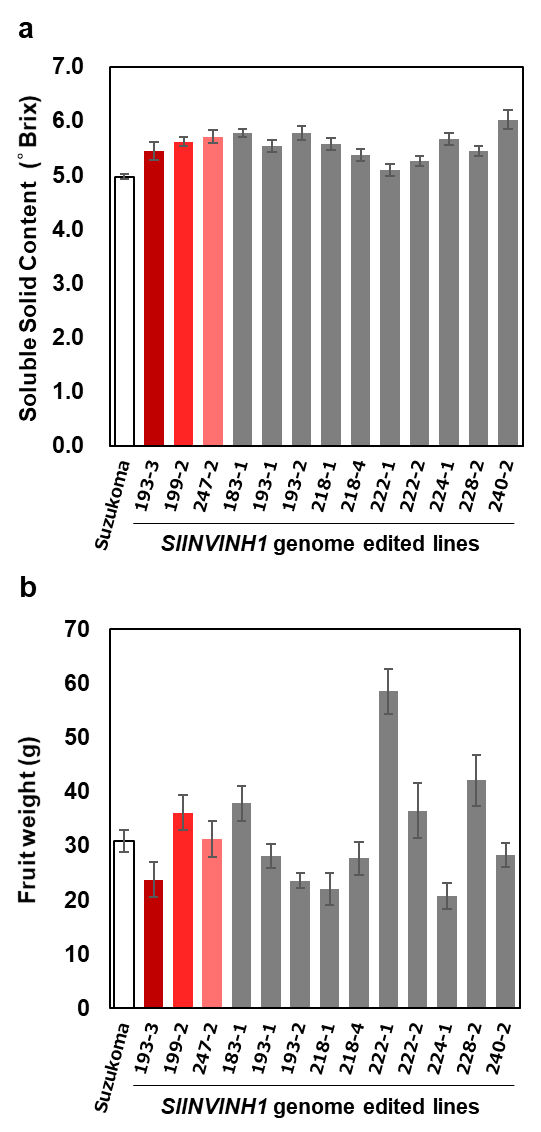
**

**Supplementary Figure S3. SSC and fruit weight of fruits from the all obtained *SlINVINH1* genome edited lines.** (a) SSC of fruits at mature stage from original cultivar ‘Suzukoma’ (white symbol) and the *SlINVINH1* genome edited lines (gray and red symbols). Red symbols indicate the selected three lines (193-3, 199-2 and 247-2) for detailed analysis. Error bars indicate standard error for more than 9 fruits. (b) Fruit weight of ‘Suzukoma’ (white symbol) and the *SlINVINH1* genome edited lines (gray and red symbols). Red symbols indicate the selected three lines (193-3, 199-2 and 247-2) for detailed analysis. Error bars indicate standard error for more than 9 fruits.


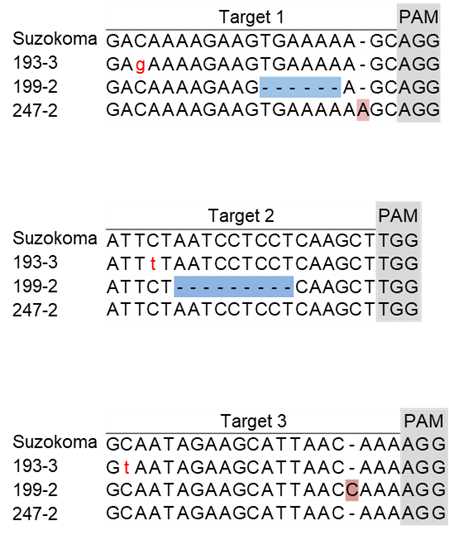


**Supplementary Figure S4.** **Mutations in *SlINVINH2* of the genome edited lines.** Target sequence in original cultivar ‘Suzukoma’ is shown at the top in each panel and the sequence in three genome edited lines (193-2, 199-2 and 247-2) are shown below. Gray shad indicate PAM sequence. Red shad, blue shad or red small letter show insertion, deletion or substitution of nucleotide(s), respectively.


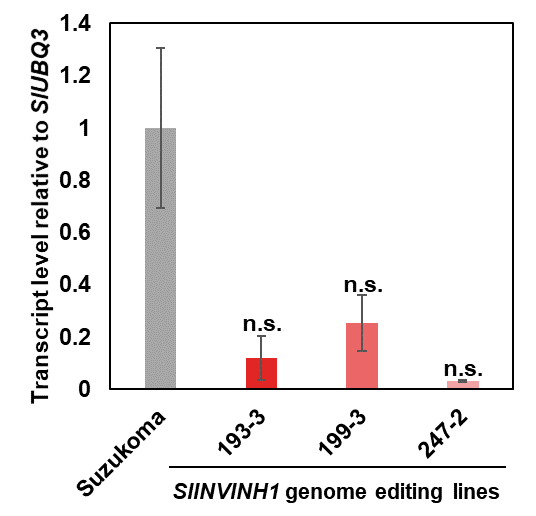


**Supplementary Figure S5. Quantitative RT-PCR analysis of *SlINVINH1* in *SlINVINH1* genome editing lines.** Quantitative RT-PCR analysis of *SlINVINH1* in mature fruit of *SlINVINH1* genome edited line and the original cultivar ‘Suzukoma’. Transcript level is shown as the value relative to *SlUBQ3* gene. Error bars indicate standard error for three biological replicates each *SlINVINH1* genome edited lines. n.s. indicates a no significant difference (p > 0.05, Dunnett’s test).


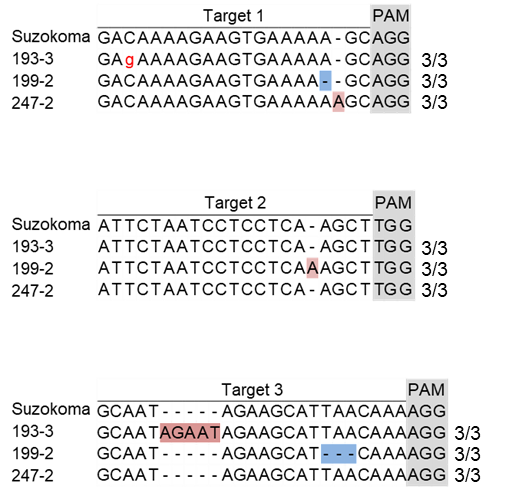


**Supplementary Figure S6.** ***SlINVINH1* transcript sequence of the *SlINVINH1* genome editing lines.** Target sequence in original cultivar ‘Suzukoma’ is shown at the top in each panel and the transcript sequence from cDNA clone in three genome edited lines (193-2, 199-2 and 247-2) are shown below. Grey shade indicates PAM sequence. Red shad, blue shad or red small letter show insertion, deletion or substitution of nucleotide(s), respectively. The right side indicate detected clone numbers in the transcript sequencing analysis.
